# Trends and complications profile in childhood-onset type 1 diabetes from North India

**DOI:** 10.1186/1687-9856-2013-S1-P10

**Published:** 2013-10-03

**Authors:** Tushar Godbole, Veena Nair, SVB Reddy, Vijayalakshmi Bhatia, Preeti Dabadghao, Kumudini Sharma, Eesh Bhatia

**Affiliations:** 1 Sanjay Gandhi Postgraduate Institute of Medical Sciences, Lucknow, India

## 

Data from India regarding epidemiology and complications of childhood onset type 1 diabetes (T1DM) is scarce. We analyzed the epidemiology, glycemic control and diabetes related complications in our T1DM patients. Relevant clinical data of patients with onset of T1DM <18 years of age attending our OPD from January 1991 till December 2010 was collected from hospital records, phone calls, letters and direct patient interviews. Patients following up between January 2010 to December 2011 were evaluated for glycemic control. Of 409 patients having at least 1 visit in our clinic over 20 years, 269 patients could be contacted in the study period. The proportion of newly registered T1DM patients among all pediatric endocrine new patients increased 2.4 times between years 2000 and 2010. The proportion of patients diagnosed at age < 5 years increased over time and was 24 % by year 2010. Premixed insulin or other unphysiological regimens were used by 65 % of patients at the time of referral. In the hospital 98 % were initiated/ shifted to one of the physiological regimens. Severe hypoglycaemia occurred in 44 % patients at least once. Prospective evaluation during the two year period was done for 187 patients. The median age at evaluation was 16 years [2-49 years] and median duration of diabetes at follow up was 5 years [0.3-28 years]. Mean HbA1c was 8.7±1.9%. ADA criteria for eligibility for complication screening were met in 106 patients. Prevalence of hypertension was 17.8% and of clinical neuropathy 16.9%. Ophthalmologic evaluation, performed in 60 patients, showed cataracts in 8.3% and diabetic retinopathy in 16.6%. Five patients had proliferative retinopathy. Microalbuminuria was present in 13.8% patients and 2.7% had gross albuminuria. Four patients had chronic renal failure. Among the 269 patients whose living status is known, there were 19 deaths (10 females). Mean age at death was 20.1 ± 8.1 years and mean duration of diabetes at death 120 ± 85 months. Six deaths were due to causes unrelated to DM. In conclusion, there is indirect evidence to suggest an increase in T1DM in younger children. Mortality is unacceptably high. A significant proportion develops diabetes related complications.

